# Catfish spine envenomation and bacterial abscess with *Proteus* and *Morganella*: a case report

**DOI:** 10.1186/1752-1947-7-122

**Published:** 2013-04-30

**Authors:** Gary Huang, Robert Goldstein, Donna Mildvan

**Affiliations:** 1 Boston Medical Center, One Boston Medical Center Place, Boston, MA 02118, USA; 2 Sound Shore Medical Center, 16 Guion Place, New Rochelle, NY 10802, USA; 3 Beth Israel Medical Center, Milton and Carroll Petrie Division, First Avenue at 16th Street, New York, NY 10003, USA

## Abstract

**Introduction:**

Abscess formation and cellulitis in the setting of envenomation are rare complications of handling catfish. To the best of our knowledge, isolation of *Proteus vulgaris* has not been previously recorded, and recovery of *Morganella morganii* has been reported in only one prior case from wound cultures in patients injured by catfish stings. We report a case of catfish envenomation characterized by abscess formation and cellulitis, in which wound cultures grew these unusual organisms.

**Case presentation:**

A 52-year-old Chinese-American man was hospitalized with erythema and swelling of his right arm of 10 days’ duration after skin penetration by a catfish barb. An abscess of his right thumb had undergone incision and drainage, with purulent drainage sent for wound culture immediately prior to admission. Laboratory studies revealed elevated white blood count, sedimentation rate, and C-reactive protein. The patient was treated with intravenous ampicillin-sulbactam and vancomycin during his hospitalization, and symptoms improved. Wound cultures obtained prior to presentation grew many *Proteus vulgaris* and *Morganella morganii*. He was subsequently discharged on a 10-day course of oral ciprofloxacin and amoxicillin-clavulanate. At a 12-month telephone follow-up, the patient denied developing further symptoms and reported that the wound had healed completely without complication.

**Conclusion:**

Although envenomation and secondary infection are not uncommon sequelae of handling catfish, the present case is unique by virtue of the infecting organisms isolated. Given the prevalence of injury from catfish stings, a review of the literature is presented in order to provide recommendations for prevention and treatment of catfish envenomation.

## Introduction

Catfish have been farmed as food for the past several hundred years throughout the world, in Africa, Asia, Europe, and North America. One of the most commonly farmed catfish in the United States of America is channel catfish, *Ictalurus punctatus*, one of more than 1000 catfish species. Virtually all catfish, including *Ictaluridae*, possess spines on their dorsal and pectoral fins, which serve as defense mechanisms when they are agitated or disturbed [1]. In addition to inducing mechanical injury, the spines also contain venom glands, which, when compressed after the overlying sheath has been broken, release venom that can cause both a severe local inflammatory reaction and possible systemic symptoms. However, the most serious complications of catfish stings in humans involve bacterial superinfections. Waterborne organisms such as *Vibrio* species for saltwater infections and *Aeromonas* for freshwater infections have been isolated from sting wounds and have been commonly reported [2,3].

By contrast, we report a case of a channel catfish spine puncture complicated by a *Proteus* and *Morganella* bacterial abscess, representing an apparently unique infectious complication of catfish injury.

## Case presentation

A 52-year-old immunocompetent Chinese-American man with no significant past medical history, including an absence of chronic diseases, was injured while handling a catfish 10 days prior to admission while working as a fishmonger in a New York City supermarket. He had picked up a live channel catfish (*I. punctatus*) from a fish tank with his ungloved right hand, after which he was stung in the right nail groove of his thumb by the spine of the catfish. The patient experienced immediate and severe pain at the puncture site. As the day progressed, he developed pain, erythema, and swelling throughout his right thumb. Over the next few days, the patient reported an increase in pain from 1 out of 10 to 7 out of 10 in intensity, with radiation to his right forearm, and progressive erythema and swelling which extended proximally up his right arm. Subsequently, he sought medical attention from his primary care physician, who found the patient to be afebrile and prescribed amoxicillin-clavulanate to treat cellulitis and ibuprofen as needed for pain control. The patient revisited his physician 3 days later with the development of an abscess and no response to the antibiotic while remaining afebrile. The ibuprofen that he was taking for pain control likely served as an anti-pyretic and obscured possible fever. His doctor performed an incision and drainage procedure of the lesion and sent the purulent drainage for wound culture. He then referred the patient to the emergency department for admission and intravenous antibiotics. There, the patient was given 900mg intravenous clindamycin and tetanus immunization, as well as ibuprofen 600mg for pain control.

**Figure 1:**
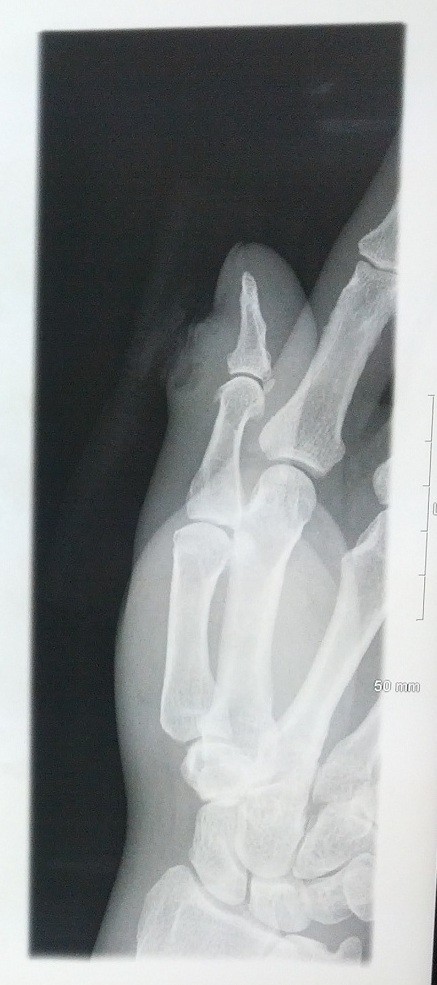
**Right thumb radiographs taken on admission to the hospital.** Plain films show cellulitis and edema of the skin overlying the interphalangeal joint of the first digit. There is no evidence of fracture, dislocation, or osteomyelitis.

Upon admission, the patient reported the pain as 2 out of 10 diffusely in his right thumb. He described the pain as throbbing and intermittent, with radiation to his right forearm. The patient was non-toxic appearing, but in severe pain. Vital signs demonstrated a temperature of 96.9°F (36.1°C), pulse of 62 beats per minute, respiratory rate of 18, and blood pressure of 112/71mmHg. The physical examination was unremarkable aside from an indurated, red, firm 2cm swelling to the medial aspect of his right thumb that was tender to palpation, with surrounding erythema and warmth, and lymphangitic erythematous streaks that tracked medially to his antecubital fossa. The laboratory evaluation was unremarkable, including normal liver and renal panels, except for an elevated white blood cell count (WBC) of 13.2K/uL (80% neutrophils), sedimentation rate of 38mm/hour (reference range 0 to 13), and C-reactive protein of 4.5mg/dL (reference range 0 to 1). X-ray views of the thumb were negative for foreign body and gas (Figure 1). There was no evidence of cortical irregularity or periosteal reaction to suggest osteomyelitis.

The patient was initially treated with intravenous tobramycin, oral tetracycline, and intravenous ampicillin-sulbactam. Hydrogen peroxide immersion of his right thumb and wet to dry dressings were used for wound care. One day after admission, the patient’s WBC decreased to 7.8K/uL, and Gram stain from the wound on initial presentation revealed moderate Gram-negative bacilli and a few Gram-positive cocci in pairs. Ampicillin-sulbactam was continued and vancomycin was added for possible methicillin-resistant *Staphylococcus aureus* coverage. After substantial relief of symptoms and reduced signs, including less erythema and induration, and normalization of the WBC, the patient was discharged and prescribed a 10-day course of oral ciprofloxacin and amoxicillin-clavulanate. Wound cultures obtained by his primary care physician grew many *Proteus vulgaris* and *Morganella morganii*. Table 1 shows the antimicrobial susceptibility data of the two case isolates. Both organisms, while susceptible to ciprofloxacin, with minimum inhibitory concentration (MIC) less than 0.25μg/mL, were resistant to ampicillin, with MIC greater than 32μg/mL. At a 12-month telephone follow-up, the patient denied developing further symptoms and reported that the wound had healed completely without complication.

**Table 1:** **Antimicrobial susceptibility test results of the two isolates, *Proteus vulgaris *** **and *Morganella morganii***

	***Proteus vulgaris***		***Morganella morganii***	
**Drug**	**MIC (μg/mL)**	**Interpretation**	**MIC (μg/mL)**	**Interpretation**
Amikacin	<2	S	<2	S
Ampicillin	>32	R	>32	R
Ampicillin-sulbactam	<2	S	>32	R
Cefazolin	>64	R	>64	R
Cefepime	<1	S	<1	S
Cefotetan	<4	S	<4	S
Ceftazidime	<1	S	<1	S
Ceftriaxone	<1	S	<1	S
Cefuroxime axetil	>64	R		
Ciprofloxacin	<0.25	S	<0.25	S
Gentamicin	<1	S	<1	S
Levofloxacin	<0.25	S		
Piperacillin	<4	S	<4	S
Piperacillin-tazobactam	<4	S	<4	S
Ticarcillin-clavulanic acid			<8	S
Tobramycin	<1	S	<1	S
Trimethoprim-sulfamethoxazole	<20	S	<20	S

*MIC* minimum inhibitory concentration; *R* resistant; *S* susceptible.

## Discussion

**Figure 2:**
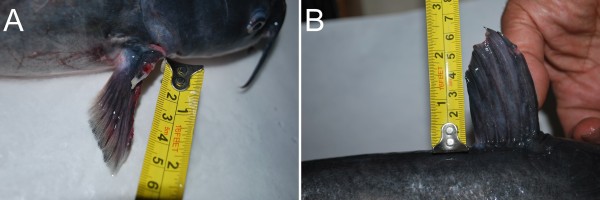
**Photographs of channel catfish, *Ictalurus punctatus*, with exposed pectoral (A) and dorsal spines (B).** Note the sharp and deeply serrated contours of the spines.

Over 1000 species of freshwater and saltwater catfish exist worldwide, with some weighing a few grams and others up to 200kg. They vary greatly in their adaptations to different ecological conditions. An Egyptian catfish, *Malapterurus*, contains electrical organs capable of causing a fatal electric shock in humans [1]. Candiru (genus *Vandellia*) is a small Amazonian catfish that is attracted to urine and may penetrate the urethral orifice of mammals, including humans, requiring surgical intervention [2]. Almost all catfish have the ability to inflict extremely painful wounds with their pectoral and dorsal spines (Figure 2). The freshwater catfish *I. punctatus* is capable of causing significant injury with its stings [1]. Contrary to popular belief, the prominent barbels (whiskers) characteristic of catfish are for sensory purposes only and are incapable of causing envenomation.

**Figure 3:**
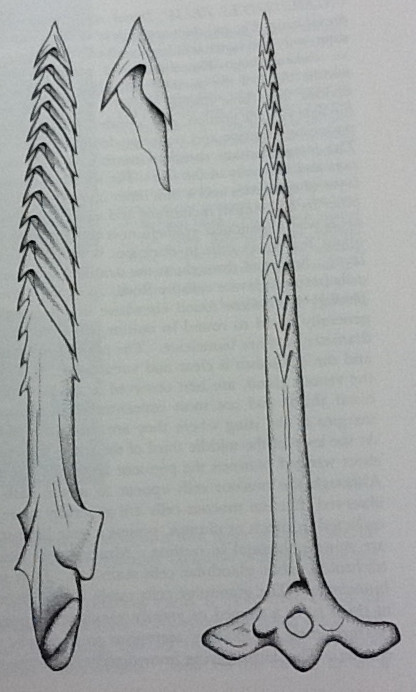
**Dorsal spine of the striped eel catfish, *Plotosus lineatus ***[1]**.** Permission for use obtained from Darwin Press, Inc.

Envenomations generally occur when the catfish are being handled. They react to being grasped by lashing from side to side and locking their dorsal and pectoral spines, which are enclosed in an integumentary sheath containing venom glands, into a rigid and extended position (Figure 3).

These sharp spines may penetrate skin, in the process damaging the delicate integumental sheath and exposing the venom glands. The retrorse barb (upturned tip) that *Ictaluridae* possess on their spines is capable of lacerating skin, facilitating absorption of the venom and often necessitating surgical removal [1]. Catfish venom consists of hemolytic, dermonecrotic, edema-producing, and vasospastic factors, all of which have shown to be heat, pH, and lyophilization labile [3]. A second source of toxins, crinotoxins, is released by the epidermal cells of catfish skin upon agitation. These proteinaceous substances may coat the spine and become passively introduced into the wound upon skin breach [4]. Both venom and crinotoxin promote a marked localized inflammatory reaction, resulting in common findings of local erythema, throbbing pain, hemorrhage, edema, cyanosis, and lymphangitis [5]. Systemic manifestations are rare, and the majority of cases resolve without long-term sequelae [6]. However, disabling sequelae including amputation of the affected body part due to severe tissue necrosis and death have been reported [7].

Although an infrequent occurrence, the most serious long-term complications of catfish envenomations involve infections. *Ictaluridae* are freshwater catfish that generally inhabit stagnant and dirty waters, potentially increasing the risk of infection. The vasoconstrictive effects of catfish toxins may also add to the infection risk by decreasing blood flow to the affected tissue [8]. A variety of organisms have been reported to be responsible for causing secondary infection, including *Klebsiella*, *Erysipelothrix*, *Nocardia*, *Chromobacterium*, *Sporothrix*, *Actinomyces*, *Pseudomonas, Staphylococcus*, *Morganella, Edwardsiella*[7], *Mycobacterium*[9], *Aeromonas*, and *Vibrio* species [7]. *Aeromonas* and *Vibrio* species have been reported to be the most aggressive organisms for freshwater and saltwater infections, respectively, especially in immunocompromised patients [6,7].

The genera *Proteus* and *Morganella* are motile, facultative anaerobic Gram-negative rods with peritrichous flagella, and are assigned to the *Enterobacteriaceae* family mainly on the basis of shared biochemical characteristics, including the ability to oxidatively deaminate phenylalanine and, in most cases, to hydrolyze urea. In human disease, most infections are associated with prolonged hospitalization and, specifically, from colonization of indwelling catheters and associated urinary tract infections [10].

Although Sarter and colleagues isolated *Proteus vulgaris* from a catfish farm in the Mekong Delta, Vietnam [11], the present case report is the first, to the best of our knowledge, to describe catfish envenomation resulting in secondary infection by *Proteus vulgaris*. Junqueira performed a microbiological evaluation of the catfish to determine the array of organisms directly isolated from the fish [12]. Of interest, neither Gram-positive bacteria nor fungi were detected in these samples, which included 13 different *Enterobacteriaceae*, the least frequent of which was *Proteus* species. In addition, whereas the aforementioned study isolated various bacterial species directly from catfish, our study demonstrates patient isolates in the setting of a clinical infection. A MEDLINE search over the past 30 years identified only two other case reports of *M. morganii* infection following catfish envenomation [13].

Effects from catfish toxins, such as pain, erythema, and edema, are difficult to differentiate from a local bacterial process. However, we suspect that our patient was infected with *P. vulgaris* and *M. morganii* secondary to catfish sting. The suspicion arose because in addition to the positive wound cultures for these organisms, the patient’s condition worsened after outpatient therapy with amoxicillin-clavulanate, to which *M. morganii* was resistant, and improved only after having received broad spectrum Gram-negative coverage with tobramycin and ciprofloxacin, which are antibiotics that target both bacteria. The persistence of local symptomatology for days into the hospital course further supports the interpretation that a bacterial infection was present because toxin-mediated symptoms are usually short-lived, whereas bacterial infections generally persist. Sources of these bacterial strains include both the catfish and its aquatic environment, as numerous bacterial species have been isolated from the water and sediment in which catfish inhabit [14].

Initial treatment of catfish envenomation should include aggressive cleaning of the wound and the surrounding area, with an attempt to remove any remnants of spinal sheath, as this radiolucent organic matter may promote inflammation and harbor virulent waterborne organisms. Plain radiographs should be done to evaluate for foreign material and gas in the wound. Initial treatment also includes prompt administration of tetanus prophylaxis and empiric antibiotics to cover *Aeromonas* and *Vibrio* strains in freshwater and saltwater accidents, respectively. The antibiotics of choice for empiric treatment of *Aeromonas* are fluoroquinolones, including ciprofloxacin and levofloxacin, due to their broad Gram-negative effects [15]. Of note, *Aeromonas* is often resistant to penicillins and cephalosporins. A recommended antibiotic regimen for empiric coverage of *Vibrio* species involves doxycycline with the addition of either ceftazidime or a fluoroquinolone. Antibiotics should be adjusted based on organisms isolated and susceptibility results. After initial management, the wound should be thoroughly cleansed, irrigated, explored, and debrided if necessary, after which the lesion should be left open. The affected extremity should then be splinted and the patient closely monitored. In our patient, the presence of healthy appearing deep tissues coupled with a progressive improvement of signs and symptoms led us to pursue a conservative approach.

Live catfish should be handled carefully with gloves to avoid accidental encounters with spines. One way to handle a live catfish out of water is to grasp it behind the pectoral fins, keeping the dorsal spine pressed down with the palm of the hand [7]. Another suggested method involves gently grasping the fish in an anterior-to-posterior direction so that the erect dorsal spine fits safely between the second and third digits [6].

## Conclusion

Catfish stings are a common occurrence among those who regularly handle catfish. Symptoms range from the localized, short-lived inflammatory effects of envenomation to the more severe systemic, long-term complications of secondary bacterial infection. Effective medical management of superficial skin infections usually only involve antibacterial coverage of Gram-positive organisms, including *Streptococcus* and *Staphylococcus*. However, superficial skin infections resulting from catfish stings are likely to be caused by Gram-negative organisms, including *Enterobacteriaceae*. Therefore, as illustrated by this case, empiric antibiotic treatment for these infections should include, in addition to Gram-positive coverage for *Streptococcus* and *Staphylococcus*, broad-spectrum Gram-negative coverage.

## Consent

Written informed consent was obtained from the patient for publication of this case report and accompanying images. A copy of the written consent is available for review by the Editor-in-Chief of this journal.

## Abbreviations

MIC: Minimum inhibitory concentration; WBC: White blood cell count.

## Competing interests

The authors declare that they have no competing interests.

## Authors’ contributions

GH was the chief author of the manuscript. RG and DM were involved in revising the manuscript critically for important intellectual content. All authors read and approved the final manuscript.
